# A Sustainable, Green-Processed, Ag-Nanoparticle-Incorporated Eggshell-Derived Biomaterial for Wound-Healing Applications

**DOI:** 10.3390/jfb14090450

**Published:** 2023-09-01

**Authors:** Rosemond A. Mensah, Federico Trotta, Emily Briggs, Nik San Sharifulden, Lady V. Barrios Silva, Zalike Keskin-Erdogan, Seyta Diop, Alvena K. Kureshi, David Y. S. Chau

**Affiliations:** 1 Division of Biomaterials and Tissue Engineering, Eastman Dental Institute, University College London, Royal Free Hospital, Rowland Hill Street, London NW3 2PF, UK; 2 Metalchemy Limited, 71-75 Shelton Street, London WC2H 9JQ, UK; 3 Department of Materials, Henry Royce Institute, The University of Manchester, Rumford Street, Manchester M13 9PL, UK; 4 Chemical Engineering Department, Imperial College London, Exhibition Rd, South Kensington, London SW7 2BX, UK; 5 Centre for 3D Models of Health and Disease, Division of Surgery and Interventional Science, University College London, Charles Bell House, Foley Street, London W1W 7TY, UK

**Keywords:** eggshell membrane, silver nanoparticles, antimicrobial activity, dermal wound healing, green process technology

## Abstract

The eggshell membrane (ESM) is a natural biomaterial with unique physical and mechanical properties that make it a promising candidate for wound-healing applications. However, the ESM’s inherent properties can be enhanced through incorporation of silver nanoparticles (AgNPs), which have been shown to have antimicrobial properties. In this study, commercially produced AgNPs and green-processed AgNPs were incorporated into ESM and evaluated for their physical, biological, and antimicrobial properties for potential dermal application. The ESM was extracted using various techniques, and then treated with either commercially produced AgNPs (Sigma-Aldrich, Poole, UK) or green-synthesized AgNPs (Metalchemy, London, UK) to produce AgNPs-ESM samples. The physical characteristics of the samples were evaluated using scanning electron microscopy (SEM), Fourier Transform Infrared (FTIR) spectroscopy, and the biological properties were assessed through in vitro studies using human dermal fibroblasts (HDFs) and BJ cells. The SEM analysis of the AgNPs-ESM samples showed localization of AgNPs on the ESM surface, and that the ESM maintained its structural integrity following AgNP incorporation. The FTIR confirmed loading of AgNPs to ESM samples. The biological studies showed that the 5 μg/mL AgNPs-ESM samples were highly biocompatible with both HDFs and BJ cells, and had good viability and proliferation rates. Additionally, the AgNPs-ESM samples demonstrated pro-angiogenic properties in the CAM assay, indicating their potential for promoting new blood vessel growth. Assessment of the antimicrobial activity of the enhanced AgNPs/ESMs was validated using the International Standard ISO 16869:2008 methodology and exploited *Cladosporium*, which is one of the most commonly identified fungi in wounds, as the test microorganism (≥5 × 10^6^ cells/mL). The AgNPs-ESM samples displayed promising antimicrobial efficacy as evidenced by the measured zone of inhibition. Notably, the green-synthesized AgNPs demonstrated greater zones of inhibition (~17 times larger) compared to commercially available AgNPs (Sigma-Aldrich). Although both types of AgNP exhibited long-term stability, the Metalchemy-modified samples demonstrated a slightly stronger inhibitory effect. Overall, the AgNPs-ESM samples developed in this study exhibited desirable physical, biological, and antimicrobial properties for potential dermal wound-dressing applications. The use of green-processed AgNPs in the fabrication of the AgNPs-ESM samples highlights the potential for sustainable and environmentally friendly wound-healing therapies. Further research is required to assess the long-term biocompatibility and effectiveness of these biomaterials in vivo.

## 1. Introduction

Wound healing has been a major concern for healthcare providers worldwide, as a result of the high prevalence of acute and chronic wounds caused by injuries, diseases, or surgical procedures, and their impact on quality of life and the economy [1,2]. In conventional therapies for skin wound healing, the primary objective is the efficient management of the wound, with a view to prevent the onset of chronicity. Conventionally, skin wound-healing therapies, such as biological and synthetic skin substitutes (skin graft), synthetic dressings, and bandages, have been recognized and employed. However, it is imperative to note that these therapies have several limitations, namely, cost, limited availability, donor site morbidity, and poor wound-healing outcomes. Thus, there has been increasing interest in developing novel biomaterials that can promote wound healing and enhance clinical outcomes [3,4,5,6].

Chicken (Gallus gallus) ESM is a natural biomaterial obtained from chicken egg and has good biocompatibility, biodegradability, antibacterial, antifungal, and wound-healing properties [7]. The resemblance of the ESM to native tissue, i.e., the extracellular matrix, and its high protein and glycoprotein content, make it a highly valued resource in wound healing and capable of providing an ideal environment for tissue healing [7,8,9]. Furthermore, the high surface area and functional groups, porosity, biological attributes, and non-toxic character make the ESM a perfect scaffold to encourage cell growth and migration [7,10]. Likewise, the high surface area of the ESM lets it serve as an adsorbent of drugs, nanoparticles, or microparticles to improve tissue regeneration via controlled drug release [7,9]. Based on these characteristics, the ESM have been exploited as a potential biomaterial for dermal wound-healing applications [7,9,11,12,13,14,15]. One important process for the use of ESM as a biomaterial is its separation from the eggshell (ES). The method of extraction plays a critical role in determining the final composition, structure, biological characteristics, and wound-healing properties of the ESM. Several reports have demonstrated approaches to extract the membrane from the ES, which include manual peeling, mechanical, enzymatic, and chemical methods [7,8,9].

The potential application of AgNPs for dermal wound-healing treatment has been extensively investigated [16]. The antibacterial, antifungal, and antiviral properties of AgNPs have been demonstrated in several studies, proving their effectiveness against a wide range of microorganisms such as bacteria, viruses, and fungi [17]. The small size of AgNPs allows them to penetrate the cell membrane of pathogenic microorganisms, disrupting their cellular functions and causing their death. Furthermore, it has been documented that AgNPs accelerate wound healing by promoting cell proliferation, angiogenesis, and collagen deposition [16,18]. The synthesis of AgNPs can be achieved via various techniques, including chemical, physical, and biological techniques [16,17,18,19,20]. The chemical approach involves the reduction of Ag^+^ ions using reducing agents, whereas the physical technique involves the use of high-energy sources such as lasers for the synthesis of AgNPs [19]. The biological approach involves the utilization of microorganisms or plant extracts to synthesize AgNPs [17]. Commercial AgNPs are widely available and are commonly synthesized using chemical methods which involve the use of hazardous chemicals with harmful environmental impacts. In contrast, green-synthesized AgNPs are produced using environmentally sustainable methods and have been documented to demonstrate superior antimicrobial activity and to be more cost effective compared to chemically synthesized AgNPs [20].

The incorporation of AgNPs into the ESM can enhance its antimicrobial/antimycotic properties through the generation of a composite material with enhanced characteristics. Moreover, the fibrous structure of the ESM can act as a scaffold for the AgNPs, allowing for a desired sustained controlled release of Ag^+^ ions over time. This can provide long-lasting antimicrobial activity against wound infections or other microbial contamination [9,18,21,22,23]. Previous research has indicated the presence of *Cladosporium* in wound infections, highlighting its potential pathogenicity and its role as an opportunistic pathogen [24,25]. Thus, in this study, ESM samples were extracted using a number of different techniques and further enhanced by incorporating either standard AgNPs that were available commercially (Sigma-Aldrich), or using novel AgNPs manufactured by means of a unique green patented process (Metalchemy, Patent No: GB2598715A). These modified samples were then profiled in terms of the physical, biological, and antimicrobial/antimycotic characteristics, with the ultimate aim of assessing their potential dermal wound-healing applications.

## 2. Materials

Free-range, large, brown chicken eggs (Light Sussex, Gallus gallus) were bought from a local supermarket (Marks and Spencers, London, UK). Fertilized hens’ eggs were purchased from Henry Stewart and Co limited (Norfolk, UK). Acetic acid (AA) (glacial, 99.7%) was purchased from Alfa Asear (36289, Heysham, UK). Penicillin/streptomycin (P/S) (P4333), human fibroblast (BJ) cells (9254, 94022524), and silver dispersion nanoparticle solution (0.02 mg/mL in aqueous buffer) (730785) were purchased from Sigma-Aldrich (Poole, UK). Green-synthesized silver nanoparticle solution (1.255 × 10^−2^ mg/mL) was obtained from Metalchemy (London, UK). Dulbecco’s modified Eagle medium (DMEM) (21331046), fetal bovine serum (FBS) (A4766801), phosphate-buffered saline (PBS, pH 7.2) (20012019), and human dermal fibroblast (HDF) (C0125C) cells were purchased from GIBCO (Thermo Fisher, Paisley, UK). CellTiter 96^®^AQ_ueous_ One Solution Cell Proliferation assay (i.e., MTS assay) (G3582) and the CytoTox 96^®^ Non-radioactive Cytotoxicity Assay (i.e., LDH assay) (G1780) were purchased from Promega (Southampton, UK).

## 3. Methods

### 3.1. Membrane Extraction

**Figure 1:**
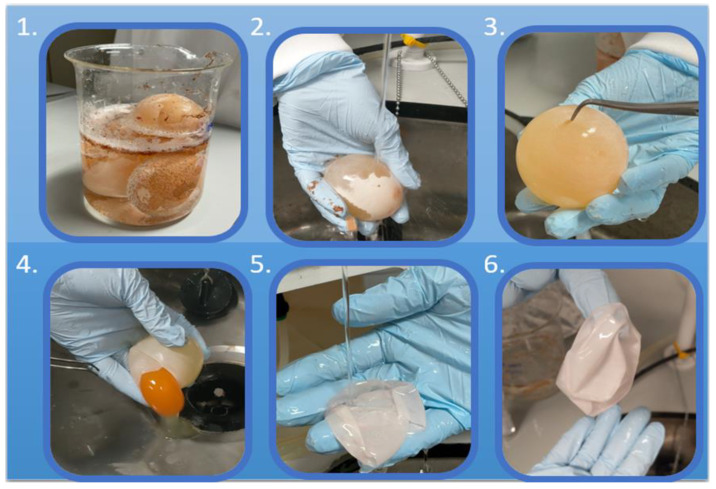
Extraction protocol of the ESM was as follows: (**1**) Eggs soaked in acetic acid for 44 h. (**2**) Washed in deionized water. (**3**) Pierced with tweezers to remove yolk and albumen. (**4**) Contents disposed of safely. (**5**) Extracted ESM sample rinsed in deionized water. (**6**) Fully extracted ESM sample (method adapted from Mensah et al. [8]).

Fresh eggs were thoroughly washed with deionized (DI) water then submerged in 0.5 M acetic acid (AA) for 44 h at room temperature (19 °C), as shown in Figure 1. After the calcium carbonated (CaCO_3_) shells were completely dissolved, the naked ESMs were thoroughly washed with DI water and pierced with a tweezer to release the albumen and yolk. The extracted ESMs were extensively washed with DI and stored in PBS to avoid dehydration (method adapted from Mensah et al. [8]). As a control, fresh eggs were manually stripped from the eggshell using tweezers.

### 3.2. Preparation of Ag-Nanoparticle-Incorporated Eggshell Membrane

**Figure 2:**
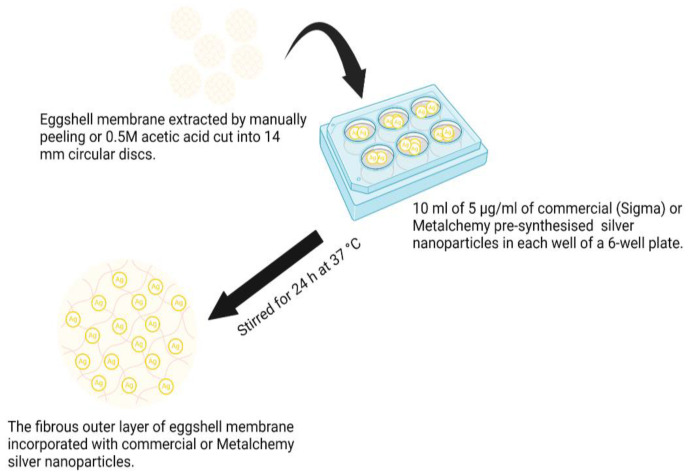
Schematic representation of the formation of Ag-nanoparticle-incorporated eggshell membrane.

The ESM samples, extracted with AA and manually peeled, were cut into circular discs using an AcuPunch^®^ (14 mm). The samples were submerged in 10 mL of 5 µg/mL of commercial AgNPs (~10 nm), used as a positive control, and green-synthesized AgNPs (~10 nm) in 6-well tissue culture plates (TCP), covered with foil and continuously stirred (50 rpm) at 37 °C for 24 h (for impregnation of AgNPs from solution to the network of ESM) [26], as observed in Figure 2. The modified samples: AA-extracted ESM incorporated with commercial AgNPs (AC) or green-synthesized AgNPs (AM) and manually peeled ESM incorporated with commercial AgNPs (SC) or green-synthesized AgNPs (SM) were air dried for 3 to 5 h before being stored at 4 °C.

### 3.3. Fourier Transform Infrared (FTIR) Spectroscopic Analysis

To investigate the biochemical makeup of the modified ESM samples (AC, SC, AM, and SM) and to verify the formation of AgNPs-ESM, FTIR spectrophotometry was utilized (Spectrum one FT-IR Spectrometer, Perkin Elmer, Llanstrisant, UK) with the Spectrum^TM^ 5 FTIR Time-based software (Perkin Elmer). An initial background scan was conducted at 37 °C for reference. Transmission (T%) was measured at wavelengths between 400 cm^−1^ and 4000 cm^−1^ at a scan speed of 0.2 cm/s (modified protocol adapted from Briggs et al. [9]).

### 3.4. Scanning Electron Microscopy (SEM) Analysis

The surface morphology of the modified ESM samples was studied using a SEM instrument (Zeiss EVO HD, Jena, Germany) operated with an accelerating voltage of 10 kV and working distance (WD) of 8.3 ± 1 mm. The 14 mm circular modified samples were fixed on conductive carbon tapes and coated with 95% gold and 5% palladium using Polaron E5000 Sputter Coater (Quorum Technologies, Laughton, UK). Images of the samples at magnification of 5000× were taken to visualize the samples (modified protocol adapted from Dubey et al. [27]).

### 3.5. AgNPs Release Study

The release profile of the modified ESM samples was examined by measuring and extrapolating the photonic resonance of AgNPs at 380 nm using an UNICAM UV 500 Spectrophotometer (Spectronic, London, UK). The 14 mm circular modified samples were placed in a 6-well plate and 10 mL of PBS (pH of 7.2) was added to each well as a release medium. The plate was agitated at about 100 rpm and incubated at 37 °C. Subsequently, 2 mL of the medium was taken at a specific time point within 14 days and replaced by fresh medium to maintain equilibrium (modified protocol adapted from Briggs et al. [9]).

### 3.6. In Vitro Cytotoxicity Study

Two different cell lines, i.e., HDF and human BJ cells, were used in this study. The HDF cells were cultured in DMEM supplemented with 10% FBS and 1% penicillin-streptomycin. The BJ cells were cultured with EDEM media supplemented with 10% FBS and 1% penicillin-streptomycin. The cell culture experiment involved using Corning T-75 flasks (Corning Life Sciences, Durham, UK) and standard trypsinisation protocol with 0.25% (*v/v*) trypsin-EDTA. The cells were cultured in a humidified incubator at 37 °C and 5% CO_2_. The 14 mm circular modified ESM samples were sterilized with UV radiation for 2 h on both sides. Afterwards, 200 µL of complete growth media were added to each sample in the well and incubated for 30 min to allow for “pre-wetting” of the membrane samples (modified protocol adapted Briggs et al. [9]).

The HDF or BJ cells were seeded on the outer sides of the blank ESM samples (controls), modified ESM samples, or TCP at a density of 2 × 10^4^ cells/cm^2^ or 10^4^ cells/cm^2^, respectively, in 200 µL of the complete growth media and incubated for one, three, seven, ten, and fourteen days under standard cell culture conditions (5% CO_2_, 37 °C). Control, TCP, and modified samples with media only were also included. Each sample group had six replicate studies.

To evaluate the metabolic activity of the cells, the CellTiter 96 AQueous One Solution Cell Proliferation assay (Promega, Southampton, UK) was used. Following one, three, seven, ten, or fourteen days of incubation, 50 µL of the culture media from the wells of three samples in each group were transferred to a new 96-well plate and retained for the LDH assay. Then, 30 µL of MTS (CellTiter96, Promega) was added to each well in the 48-well plate and incubated at 37 °C for 90 m. Next, 100 μL of spent media from each well was transferred into a 96-well plate (Corning) and repeated three times per condition. Absorbance was read at 490 nm on the Infinite M200 (Tecan, Zürich, Switzerland) plate reader. Relative metabolic activity (cell viability) was normalized to the control and calculated using the formula below, with (−) referring to the negative control, i.e., untreated cells (Equation (1)).

(1) $$\mathrm{Cell}\;\mathrm{viability}\;\left(\%\right)=\frac{\mathrm{Sample}\;\mathrm{absorbance}-\mathrm{blank}\;\mathrm{absorbance}}{\mathrm{Control}\;\left(-\right)\mathrm{absorbance}-\mathrm{blank}\;\mathrm{absorbance}}\times 100$$

A quantity of 50 μL of Triton-X lysis buffer was added to one control sample to produce a negative control and incubated at ambient temperature for 20 m; once elapsed, 50 μL of stop solution was added to arrest lysis. A quantity of 50 μL of spent media from each well was transferred to a Corning 96-transparent-well plate and repeated measures were performed in triplicate per condition. A quantity of 50 μL of lactate dehydrogenase (LDH) substrate (CytoTox96, Promega) was added to each well and incubated at room temperature for 30 m. Absorbance was then read at 490 nm using the Infinite M200 (Tecan) plate reader. Relative LDH release (cytotoxicity) was calculated as a percentage relative to the positive control (+), cells treated with Triton-X-100 (0.015 *w/v*) only, using Equation (2).

(2) $$\mathrm{Cytotoxicity}\;\left(\%\right)=\frac{\mathrm{Sample}\;\mathrm{absorbance}-\mathrm{control}\;\mathrm{absorbance}}{\mathrm{Control}\;\left(+\right)\;\mathrm{absorbance}-\mathrm{blank}\;\mathrm{absorbance}}\times 100$$

### 3.7. Antimicrobial/Antimycotic Analysis

The spore suspensions of *Cladosporium sphaerospermum* were obtained following the ISO 16869-2008 procedure [28]. The surface of the sporulating culture was gently scraped using a sterile inoculation needle and transferred to a 5 mL wetting-agent solution. After shaking the culture tube to disperse the spores, the liquid was poured into a sterile conical flask containing glass beads. This process was repeated three times with the same culture tube and conical flask. The spore suspension was then mixed with the glass beads, filtered through a thin layer of sterile cotton to remove mycelial fragments, and subjected to aseptic centrifugation. The resulting residue was washed, resuspended in a nutrient-salt solution, and centrifuged again. The washed residue was finally suspended in the same solution. The individual spore suspensions were stored at −18 °C for up to 2 months until required.

Antimicrobial activities of the AA-extracted eggshell membrane and manually peeled eggshell membrane separately incorporated with the green-synthesized AgNPs or commercial AgNPs (positive control) were assessed via the methodology presented in the International Standard ISO-16869:2008 [28]. The samples were sterilized with UV radiation for 2 h. The antimicrobial activities of the modified samples were analyzed against the test organism, *Cladosporium sphaerospermum* of domestic origin, with initial concentration of ≥5 × 10^6^ spores/mL. The 14 mm circular discs of the AgNP-incorporated membranes were placed in sterilized petri dishes containing nutrient agar medium, and 10 μL of the spore suspension was added to the center of the plate. The plates were then incubated in a Sciquip Incubator S-Series SQ-4615 at 23 ± 1 °C for 21 days. The diameter of the inhibition zone was measured using a ruler on day 1, 3, 5, 10, and 21 of incubation. Images of each petri dish were taken at regular intervals and the inhibition zone was tracked using ImageJ software (ImageJ, v1.54d, National Institutes of Health, New York, NY, USA).

### 3.8. Angiogenic Profile Analysis

The study utilized the in ovo chicken embryo chorioallantoic membrane (CAM) assay to evaluate the ability of the modified ESM samples to induce angiogenesis. A modified version of the protocols of Mensah et al. [8] and Chau et al. [29] was employed. Fertilized Dekalb White chicken eggs were incubated for four days and then a small amount of egg white was removed on the fourth day. A square window was cut in each egg, and the eggs were incubated for an additional day. On the fifth day, the sterilized modified samples were placed on the CAM. For negative controls, a blank CAM, a sterilized filter membrane (FM) on the CAM, 10 ng/mL vascular endothelial growth factor (VEGF) loaded in FM on the CAM, and blank ESMs without AgNPs were used. The eggs with all the samples were sealed and kept in the incubator for five more days. On the tenth day, the seal was removed, and images were taken and analyzed using the IKOSA Prisma CAM Assay application to quantify and characterize the blood vessels’ branching points.

### 3.9. Data Analysis

Data with replicates were statistically analyzed using PRISM Pro, version 9.0, GraphPad, Dotmatics) software. A one-way or two-way analysis of variance (ANOVA) with Tukey’s multiple comparison tests was carried out to compare sample means and variance to negative controls. Results with *p* < 0.05 (*), *p* < 0.01 (**) were considered statistically significant.

## 4. Results and Discussion

The study aimed to investigate the potential of using ESM as a carrier for AgNPs and compared the efficacy of a commercially available AgNP positive control with AgNPs synthesized using the Metalchemy green method. The ESM samples were extracted using 0.5 M acetic acid and manually stripped. The modified samples, i.e., AC or green-synthesized AgNPs (AM) and manually stripped ESM incorporated with commercial AgNPs (SC) or green-synthesized AgNPs (SM) were obtained.

### 4.1. Fourier Transform Infrared Spectroscopy

**Figure 3:**
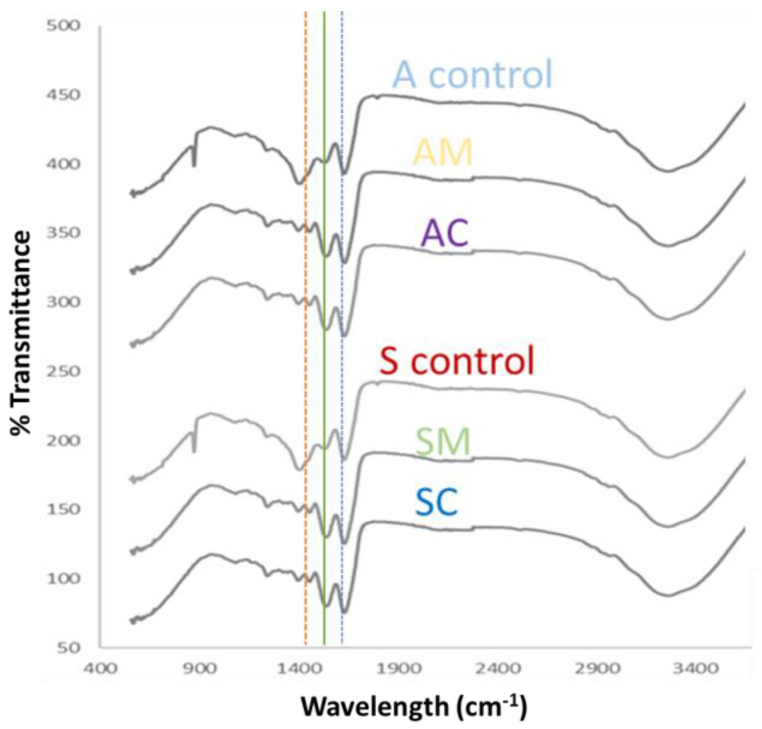
FTIR spectra of blank extracted eggshell membrane, i.e., A control and S control (unmodified ESM samples) and modified ESM samples i.e., AC, AM, SC, and SM (see abbreviation table for full details of samples). Green and blue coloured lines correspond to 1620 cm^−1^ and 1520 cm^−1^ bands, respectively. The orange line is associated with the 1400 cm^−1^ band that suggests the presence of AgNPs and their interaction with the ESM by deformations in the C-H bond.

FTIR analysis was performed to investigate if the loading of AgNPs led to chemical changes in ESM samples. The FTIR spectra of the negative control samples (A control and S control) and modified ESM samples (AM, AC, SM, and SC) were compared and are shown in Figure 3. The control samples exhibited characteristic peaks at 1620 cm^−1^ and 1520 cm^−1^, corresponding to the amide I and amide II bands of the protein component in the ESM, annotated with green and blue lines in Figure 3. The AgNP-modified ESM samples showed a shifted peak at around 1400 cm^−1^, annotated with the orange dashed line in Figure 3, which could be attributed to the presence of AgNPs and possible interaction between the ESM and AgNPs causing vibrations from the deformation of C-H bonds in the ESM [30,31,32] This could be attributed to the electrostatic attraction between the negatively charged ESM surface and positively charged AgNPs [33]. Interestingly, the ~900 cm^−1^ peak seems to be absent on the control samples, thus potentially confirming the incorporation of the AgNPs as peaks within this range corresponding to a hydroxyl group [34]. Such a disappearance following the addition of the AgNPs could be due to the formation of an ether bond [35].

### 4.2. Scanning Electron Microscopy

**Figure 4:**
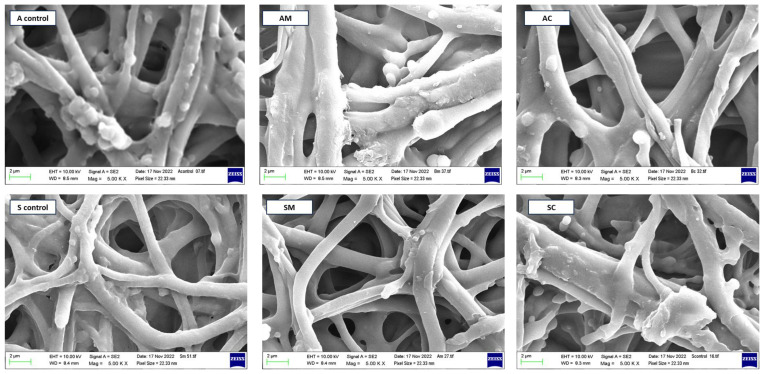
Scanning electronic microscopic images of blank extracted eggshell membrane, i.e., A control and S control and modified ESM samples, i.e., AC, AM, SC, and SM (see abbreviation table for full form of A control, S control, AC, AM, SC, and SM) visualized with a Zeiss EVO HD scanning electronic microscope (Jena, Germany) at magnifications of 5000×.

The SEM analysis was conducted to evaluate the surface morphology and structural changes in ESM samples extracted with AA and manually peeled, as well as the modified loaded ESM samples, i.e., A, SC, AM, and SM. From Figure 4, the SEM images show that the ESM extracted with AA (A control) had a more uniform and intact surface compared to the manually peeled ESM (S control), which exhibited some degree of surface roughness and irregularity. The AM and SM showed a more uniform and compact distribution of AgNPs on the surface compared to AC and SC, which appeared to be more clustered and unevenly distributed. This could be attributed to the good functional stability of green-synthesized AgNPs described in the previous literature, which could contribute to the differences in the distribution on the ESM surface seen in this study [36]. It can also be observed that the formulation method did not affect the structural intergrity of the membrane fibers.

### 4.3. Release Profile of Ag-Nanoparticle-Incorporated Eggshell Membrane

**Figure 5:**
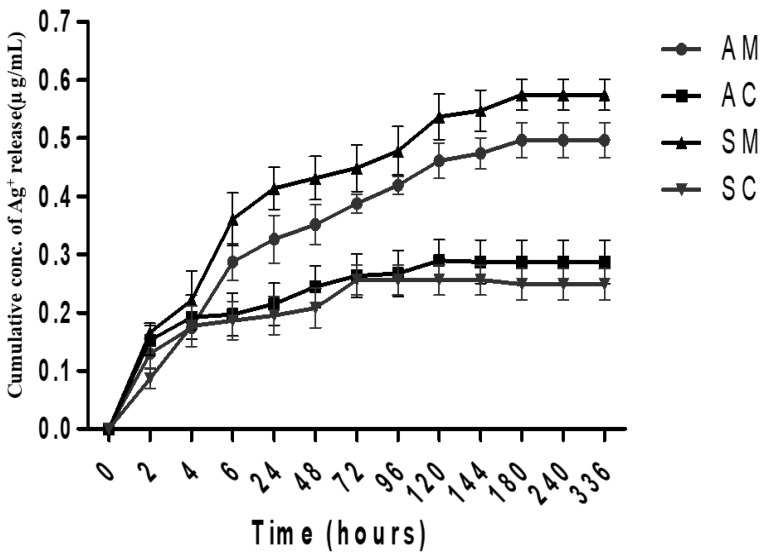
In vitro silver-ion release profile of the modified ESM samples, i.e., AC, AM, SC, and SM (see abbreviation table for full form of AC, AM, SC, and SM) at a physiological pH of 7.2 in a PBS buffer solution for 336 h (14 days) (mean ± SD, *n* = 3).

The release profile of ESM modified with either green-synthesized or commercial AgNPs was evaluated at a physiological pH of 7.2 in a PBS buffer solution over a 336 h period (14 days). The quantity of AgNPs released from the modified ESM samples was evaluated by measuring the absorbance via a UNICAM UV 500 Spectrophotometer (Spectronic, London, UK) [9]. Figure 5 shows that all four types of membranes (AM, AC, SM, and SC) released AgNPs into the PBS buffer solution, with varying degrees of release over time. The data indicate that all samples demonstrate a similar trend of AgNP release over time, with the amount of release increasing up to 120 h and then remaining relatively constant until the end of the 336 h period.

The SM showed the highest AgNP release at all time points, whereas the manually stripped eggshell membrane incorporated with commercial SC exhibited the lowest AgNP release. Statistically, no significant differences were observed between AM vs. SM (*p* > 0.05) and AC vs. SC (*p* > 0.05) (Figure 5). For AM vs. AC, the quantity of AgNP release from time point 0 h to 72 h was not significantly different (*p* > 0.05) and a significant difference was observed at 336 h (*p* < 0.001). On the other hand, no significant difference was observed between SM and SC for time point 0 to 4 h (*p* > 0.05), but a significant difference was observed up to 336 h (*p* < 0.001). Furthermore, a significantly higher quantity of AgNPs was detected to be released from the AM and SM samples in comparison to the AC and SC samples. The released quantity of AgNPs from the AM and SM samples was observed to be twice as much as that of the AC and SC samples. The identified differences in AgNP release between green-synthesized and chemical AgNPs may be explained by the difference in synthesis methods used, but demonstrate similar profiles to those previously reported [9,37].

Sustained release of Ag^+^ ions is essential for wound healing that aims to provide long-lasting antibacterial protection and is, in this application, desirable. However, it is noteworthy that while the sustained release of AgNPs is a benefit that is particularly relevant for medicine, this may not be the case for other applications and materials, and their behavior will differ accordingly depending on material composition and integration of the AgNPs [19,21,33,38]. In addition, the efficacy of AgNPs against microorganisms is not only dependent on the quantity of AgNPs released, but also on other factors such as the size, shape, and surface chemistry of the AgNPs, as well as the type of microorganism targeted. Although a prolonged release of Ag^+^ ions can help prevent the growth and spread of microorganisms in the wound, excessive AgNP release can also cause toxicity and harm to the surrounding tissue [23,39]. Hence, it is important to regulate the release of AgNPs in order to achieve effective wound healing and maintain patients’ safety. Further studies are required to investigate the ideal release profile for different applications.

### 4.4. In Vitro Cytotoxicity

**Figure 6:**
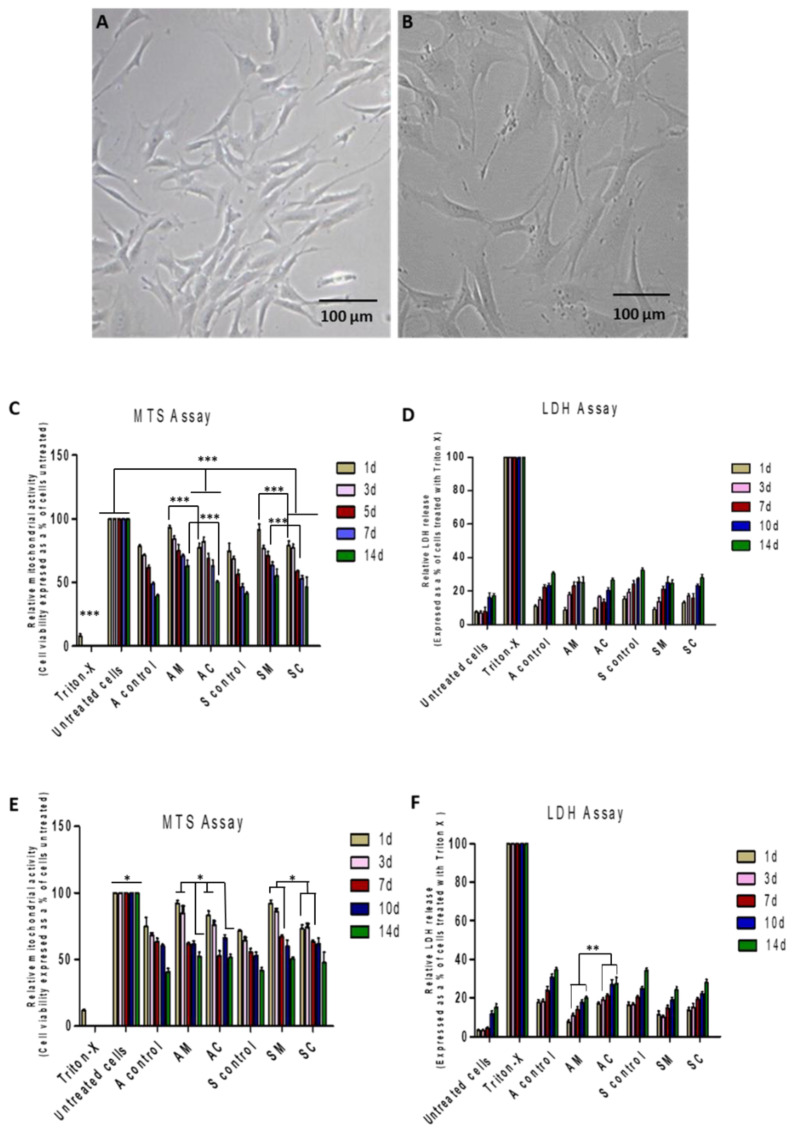
The in vitro cytotoxicity test of human dermal fibroblast (**A**) and human BJ fibroblast cells (**B**) after exposure to blank eggshell membrane (A control and S control) and modified ESM samples (AC, SC, AM, and SM) for 14 days. The relative cell mitochondrial activity (**C**,**E**) and relative LDH release (**D**,**F**) of HDF and BF cell lines cultured on treated samples and controls. See abbreviation table for full form of A control, S control, AC, AM, SC, SM, MTS, and LDH assay. (Mean ± SD, *n* = 6), (* *p* < 0.05, ** *p* < 0.01, *** *p* < 0.001).

Cytotoxicity testing is crucial for wound healing, as it ensures that the wound dressings and drugs used are safe for living cells and tissues [8,9]. In this study, MTS and LDH assays were used to determine the metabolic activity and cell death caused by modified ESM samples, which were applied to HDF (Figure 6A) and BJ (Figure 6B) cell lines. The findings revealed that the percentage of cells that exhibited viability under the treatments (AM, AC, SM, and SC) varied depending on the treatment and time point.

For the HDF cell line (Figure 6C), Triton-X treatment significantly reduced cell viability compared to the untreated group, blank samples (A control and S control), and modified samples (AC, AM, SC, and AM) at all time points (*p* < 0.001). The percentage of live cells in A control, S control, AC-treated, or SC-treated cells was observed to be significantly lower across all the time points, i.e.,1 day, 3 days, 5 days, 7 days, and 14 days (*p* < 0.001) when compared to the untreated cells. In addition, the untreated cells were significantly different (*p* < 0.001) from the cells treated with AM or SM at all time points except day 1 (*p* > 0.05). Both AM and SM exhibited higher cell viability in comparison to AC and SC at various time points (1 day, 3 days, 5 days, 7 days, and 14 days) in the HDF cell line. Specifically, the differences in cell viabilities between AM and AC were statistically significant on day 1 (*p* < 0.001) and day 14 (*p* < 0.05), whereas there were significant differences between SM and SC on day 1 and day 5 (*p* < 0.05).

LDH is a cytoplasmic enzyme that is released into cell culture media upon the damage of plasma membrane; hence, measuring LDH activity is a useful indicator of cell death (apoptosis) [40,41]. The percentage of LDH activity relative to the Triton-X control for the HDF cell line is represented in Figure 6D. Figure 6D shows that the modified ESM samples have varying effects on HDF cell viability over time. When comparing AM to SM and AC to SC, no significant differences in LDH release were observed over the 14-day period (*p* > 0.05). A strong correlation between cell viability and LDH release was discovered, with similar findings for Triton X or untreated cells compared to the blank control or modified samples. However, a weaker correlation was observed for AC vs. AM and SC vs. SM.

For BJ cells (Figure 6E), when comparing untreated cells to all other samples, the cell viability results showed significant differences at all time points. For AM vs. AC, there was a significant increase in cell viability for AM at all time points except for day 7 (*p* > 0.05). A significant increase in cell viability was observed for SC at 3 days, 7 days, and 10 days time points (*p* < 0.05) when comparing AC to SC. For SM vs. SC, there was a significant increase in cell viability for SM at all time points except for 14d. Finally, when comparing AM to SM, there was a significant increase in cell viability for AM at the 14d time point (*p* < 0.05).

The relative LDH release study using BJ cells is presented in Figure 6F. Triton-X treatment consistently resulted in 100% LDH release, indicating complete cell lysis. Comparing AC to AM, AC had significantly higher LDH release than AM at all time points (*p* < 0.01) except for day 1 (*p* > 0.05). In contrast, no significant difference was observed between SC and SM (*p* > 0.05), or between AC and SC (*p* > 0.05). Significant differences were observed on day 1 and day 14 (*p* < 0.05) for AM and SM. There was a correlation between the MTS assay and LDH assay results, indicating that the MTS assay provided a reliable measure of cell viability, while the LDH assay was a reliable measure of cell death.

The results of this study suggest the modification with AgNPs can improve the cytotoxicity profile of ESM samples, with green-synthesized samples (AM and SM) showing the most promising results and having lower cytotoxicity profiles compared to commercial nanoparticles. However, further studies are needed to fully understand the mechanisms of cytotoxicity involved and to evaluate the long-term safety and efficacy of these modified ESM samples for wound-healing applications.

Researchers have previously explored various uses for eggshell membranes, including their potential as carrier materials for nanoparticles [21,22,33]. As such, silver nanoparticles (Ag nanoparticles) are known for their antimicrobial properties and have been widely used in various applications, including medicine, electronics, and environmental remediation. However, their cytotoxicity is a concern, as they can cause damage to cells and tissues. Using eggshell membrane as a carrier material for Ag nanoparticles may offer several potential advantages, for example, the fibrous structure of the eggshell membrane [9] provides a large surface area for the attachment of nanoparticles. This can aid with the stability and dispersibility of the nanoparticles and prevent their aggregation, which can substantially affect their biological activity. Moreover, the protein components of eggshell membrane may have the ability to interact with the nanoparticles and modify their properties, such as potentially forming a protective layer around the nanoparticles to reduce their direct contact with cells and minimizing their cytotoxic effects. Furthermore, the unique composition of the eggshell membrane, particularly the presence of glycosaminoglycans, may have the ability to scavenge reactive oxygen species (ROS) [42] and provide antioxidant properties. This could potentially counteract the oxidative stress induced by Ag nanoparticles and reduce their cytotoxicity. While the potential of eggshell membrane as a carrier material for Ag nanoparticles is promising, further research is needed to fully understand its effectiveness and mechanisms of action. Factors such as nanoparticle size, concentration, and surface coating, as well as the specific characteristics of the eggshell membrane, should be taken into consideration.

In short, the use of eggshell membrane as a carrier material for Ag nanoparticles could exhibit potential in moderating the cytotoxicity associated with Ag nanoparticles. Its fibrous structure, bioactive components, and potential antioxidant properties make it an interesting candidate for further investigation in the field of nanoparticle-based applications.

### 4.5. Antimicrobial/Antimycotic Study

**Figure 7:**
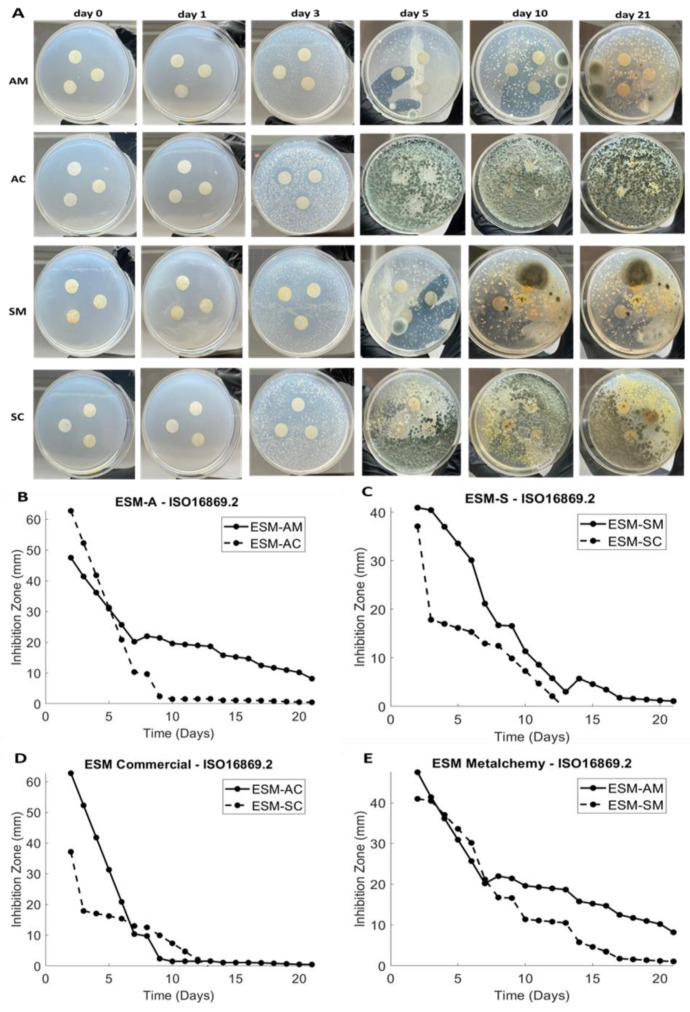
Antimicrobial activities of modified ESM samples. (**A**) Images of inhibition zones against *Cladosporium* (domestic origin) using 14 mm circular discs of AM, AC, SM, and AC incubated in a Sciquip Incubator S-Series SQ-4615 at 23 ± 1 °C at days 0, 1, 3, 5, 10, and 21. (**B**–**E**) Diameter changes of the inhibition zones of the fungi coating over 21 days following IOS 16869.2008 for the average of samples AM vs. AC, SM vs. SC, AC vs. SC, and AM vs. SM. See the abbreviation table for full form of AC, AM, SC, and SM. Images were analyzed via ImageJ software (mean ± SD, n = 3).

The antimicrobial activities of the green-synthesized AgNPs and commercial AgNPs incorporated into AA ESM and manually peeled ESM were investigated against the fungi *Cladosporium*, of domestic origin, using the International Standard ISO-16869:2008 [28]. Figure 7A illustrates the images of the inhibition zones of the fungi over 21 days.

### 4.6. Angiogenic Properties

To allow comparison between the different sizes of the zone of inhibition among samples tested against the same competitor strain, observations of changes in the quantity of antimicrobial activity from the test sample, the ability of the antimicrobial inhibitor to spread through the media, the type of AgNPs used, and the resistance level of the fungi to the AgNPs’ antimicrobial activity can be considered. Alternatively, when comparing the size of the zone of inhibition among different microbes tested against the same sample composition, assessment of the levels of resistance of the microbes to the technology used should be utilized. In short, the results up to day 10 demonstrated that Metalchemy AgNPs performed better than commercial AgNPs in all extraction methods. Moreover, after incorporation of other organisms, the green-synthesized AgNPs were shown to inhibit the growth of *Cladosporium* more effectively, resulting in populations of more resistant mold growing due to the lack of competition from *Cladosporium* when compared to commercial AgNPs.

It has been reported that *Cladosporium* is one the most abundant fungal species in the chronic wound microbiome, especially on diabetic ulcers, so that was selected as the model fungi for the antimicrobial/antimycotic study presented here. In terms of specific extraction methods, extraction using AA resulted in the greatest inhibition zone of 8.2 mm for Metalchemy AgNPs and 0.48 mm for commercial AgNPs at day 21 (AM vs. AC, Figure 7B). Metalchemy AgNPs were found to enable a 17-times greater zone of inhibition than commercial AgNPs, an indication on enhanced antimicrobial/antimycotic behavior. However, it was observed that commercial AgNPs demonstrated a stabilized inhibition zone earlier, at 9 days, while green-synthesized AgNPs displayed a steady decrease in the inhibition zone after day 7.

Overall, the study indicated that the green-synthesized AgNPs are superior in inhibiting the growth of *Cladosporium* compared to commercial AgNPs and have greater antimicrobial efficacy. The presence of phytochemicals and biomolecules in the plant extracts used in green synthesis has been reported to enhance the bioactivity of AgNPs, including their antimicrobial properties [43]. These results are consistent with previous studies where chitosan-based films with laponite and silver nanoparticles showed significant inhibition against *Aspergillus niger* and *Penicillium citrinum* [44].

**Figure 8:**
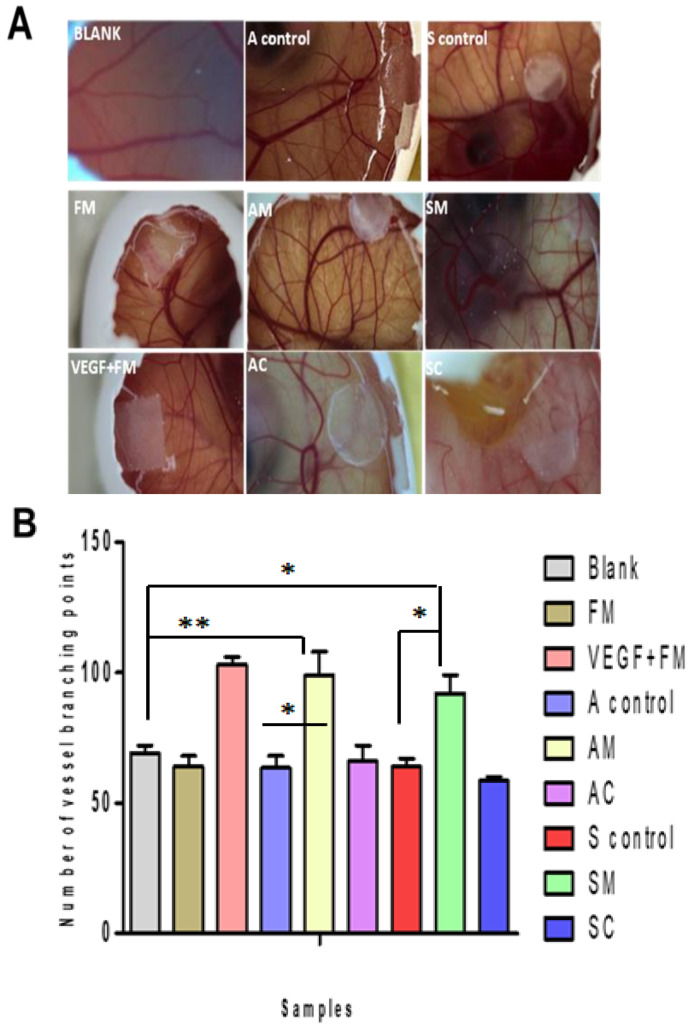
Angiogenic profile of modified samples using the chorioallantoic membrane (CAM) assay. (**A**) Photographs of CAM with no sample, i.e., blank (control) and samples i.e., FM, VEGF + FM (positive control), A control, S control, AM, AC, SM, and SC after 10 days of incubation. (**B**) Graph of the number of vessels’ branching points analyzed using the IKOSA Prisma CAM Assay application. One-way ANOVA analysis using Dunnett’s multiple comparison tests, *p* > 0.05. * represents *p* < 0.05 and ** *p* < 0.01. (mean ± SD, n = 3). See abbreviation table for full form of FM, VEGF + FM, A control, S control, AM, AC, SM, and SC.

The angiogenic profile of modified ESM samples was evaluated using the chorioallantoic membrane (CAM) assay. The CAM assay is a well-established model for studying angiogenesis and has been used to evaluate the pro-angiogenic potential of various materials, including ESM and nanoparticles [8,9,29,45,46,47,48]. The images of CAM with no sample (control) and samples after 10 days of incubation are shown in Figure 8A. The images were analyzed with the IKOSA Prisma CAM Assay application to determine the number of vessels’ branching points. As shown in the graph of the number of vessels’ branching points (Figure 8B), the VEGF+FM (positive) control had the highest number of branching points, indicating a strong pro-angiogenic effect. Among the modified samples, the AM showed a significant increase in the number of branching points compared to the negative control (blank, *p* < 0.01).

The results of the angiogenic profile analysis using CAM assay showed that the incorporation of both commercial AgNPs and green-synthesized AgNPs into the eggshell membrane significantly increased the number of vessels’ branching points compared to the negative control (blank) and the ESM controls (A control and S control) (*p* < 0.01, *p* < 0.05, and *p* < 0.05, respectively). Moreover, the comparison between the commercial AgNPs and the green-synthesized AgNPs for both extraction methods showed that the latter were more effective in promoting angiogenesis (AM vs. AC and SM vs. SC, *p* < 0.05). However, there was no significant difference observed between the ESM samples without AgNPs (A control and S control, *p* > 0.005) and the extraction method, i.e., AM vs. SM and AC vs. SC (*p* > 0.005). Additionally, no significant differences were observed in pro-angiogenic response between the green-synthesized AgNP-loaded ESM (AM and SM) and the positive control, VEGF+FM (*p* > 0.005).

The high angiogenic response of the green-synthesized AgNPs, as demonstrated by the CAM assay results, is of great significance for wound healing. Angiogenesis is an essential stage in wound-healing therapy as it involves the formation of new blood vessels that convey oxygen and nutrients to the damaged area, which in turn promotes tissue regeneration and repair. The more effective the angiogenic response, the faster and more efficient the wound-healing therapy [8,49,50,51,52]. Some studies have reported that green-synthesized AgNPs can enhance angiogenesis as compared to chemical synthesized AgNPs, while others have shown inhibitory effects on angiogenesis, and vice versa [52,53,54]. The studies suggest that the pro-angiogenic potential of AgNPs may depend on various factors such as their size, shape, and surface charge, and further research is needed to fully understand the mechanism and effects of green-synthesized AgNPs on angiogenesis.

In their recent work, Lakkim et al., 2023 noted that green-synthesized silver nanoparticles (AgNPs) derived from *Catharanthus roseus* (CAgNPs) and *Azadirachta indica* leaf extracts (AAgNPs) exhibited potent antioxidant properties in wound healing in in vivo experiments on BALB/c mice. Results showed improved wound healing with increased hydroxyproline, DNA, and protein levels in CAgNP and AAgNP-treated samples, as well as reduced scar width, fewer inflammatory cells, well-developed collagen fibers, capillaries, and fibroblasts in histological analysis [55].

In this work, the incorporation of Metalchemy green-synthesized AgNPs into the ESM was found to exhibit a pro-angiogenic effect similar to that of VEGF, a well-known and potent pro-angiogenic factor. This finding is noteworthy, as VEGF has been widely studied and utilized as a positive control in many angiogenesis assays [56,57,58,59]. The ability of the sample to produce a similar effect as that of VEGF indicates the potential of this approach as a viable option for promoting angiogenesis.

## 5. Conclusions and Future Directions

The study compared the efficacy of green-synthesized AgNPs using the Metalchemy patented method and commercially available AgNPs for wound healing and antimicrobial applications. FTIR and SEM analyses were conducted to investigate the chemical and physical changes in the ESM samples after modification with AgNPs. The release profile of ESM with green-synthesized or chemical AgNPs was examined, and it was observed that all samples released Ag+ with varying degrees of release over time. Ag^+^ release is desirable in medical applications via material design and AgNP incorporation. The differences in Ag^+^ release between Metalchemy and chemical AgNPs may be attributed to the synthesis methods used, with green-synthesized AgNPs having an improved desirable release of AgNPs compared to commercial alternatives. Sustained AgNP release is beneficial and desirable for medical applications to promote wound healing. It is also important to note that release profiles differ highly depending on material composition, end-use, and integration of the AgNPs [19].

This study demonstrated that modified ESM samples containing AgNPs have varying effects on cell viability and cytotoxicity, depending on the treatment, i.e., the type of ESM samples and time point. The MTS assay provided a reliable measure of cell viability, while the LDH assay was a reliable measure of cell death. Metalchemy samples (AM and SM) showed the most promising results in terms of lower cytotoxicity profiles/cell growth. The study found that green-synthesized AgNPs were more effective than commercial AgNPs in inhibiting the growth of *Cladosporium* fungi. Metalchemy AgNPs had a 17-times larger inhibition zone compared to commercial AgNPs (Sigma). ESM loaded with green-synthesized AgNPs were found to be more effective in promoting angiogenesis than commercial AgNPs and exhibited a pro-angiogenic effect similar to that of VEGF.

The study comparing the efficacy of green-synthesized AgNPs using the Metalchemy method and commercially available AgNPs for wound-healing and antimicrobial applications is an important contribution to the development of sustainable and effective alternatives to traditional commercial AgNPs. More research is warranted to fully explore the potential of green-synthesized AgNPs using the Metalchemy method. Future studies could investigate the optimal synthesis conditions for green-synthesized AgNPs to achieve maximum efficacy in wound-healing and antimicrobial applications. This could involve varying parameters such as temperature, pH, and response time to determine the effect on AgNPs’ morphology, size, and release profile. In addition, studies could investigate the long-term stability of green-synthesized AgNPs in biomedical applications. This would involve examining the degradation and release profile of AgNPs over an extended period to determine the potential for toxicity or adverse effects. Moreover, studies are needed to fully understand the mechanisms of cytotoxicity involved and to evaluate the long-term safety and efficacy of these modified ESM samples for wound-healing applications, e.g., enhanced in vitro studies, scratch and migration assays, and in vivo studies. Likewise, further studies could investigate the potential of green-synthesized AgNPs in other biomedical applications beyond wound-healing and antimicrobial applications, e.g., in cancer therapy, as AgNPs have been shown to have anticancer properties. Overall, the study highlights the potential of green-synthesized AgNPs as a sustainable and effective alternative to conventional commercially available AgNPs. Further investigation in this area could lead to the development of exciting novel biomedical applications and therapies.

## Data Availability

Data will be made available on request.

## Abbreviations

ESM	Eggshell membrane
AgNPs	Silver nanoparticles
C	Commercially produced silver nanoparticles (Sigma-Aldrich)
M	Green-synthesized silver nanoparticles (Metalchemy)
AgNPs-ESM	Silver nanoparticles loaded eggshell membrane
A control	Blank acetic acid extracted eggshell membrane
S control	Manually peeled eggshell membrane
AC	Acetic acid extracted ESM incorporated with commercial AgNPs
AM	Acetic acid extracted ESM incorporated with Metalchemy AgNPs
SC	Manually peeled ESM incorporated with commercial AgNPs
SM	Manually peeled ESM incorporated with Metalchemy AgNPs
FM	Filter membrane
VEGF	Vascular endothelial growth factor
VEGF+FM	FM loaded with 10 ng/mL vascular endothelial growth factor
SEM	Scanning electronic microscopy
FTIR	Fourier Transform Infrared Spectroscopy
UV	Ultraviolet
CAM	Chorioallantoic membrane
AA	Acetic acid
HDFs	Human dermal fibroblast cells
BJ	Human fibroblast cells
FBS	Fetal bovine serum
DMEM	Dulbecco’s Modified Eagle’s Medium
PBS	Phosphate-buffered saline
DI	Deionized water
MTS assay	CellTiter 96^®^ AQueous One Solution Cell Proliferation assay
LDH assay	CytoTox 96^®^ Non-radioactive Cytotoxicity Assay
P/S	Penicillin/streptomycin
EDTA	Ethylenediaminetetraacetic acid
TCP	Tissue culture plate
ANOVA	Analysis of variance
